# Is serum PSA a predictor of male hypogonadism?

**DOI:** 10.20945/2359-3997000000356

**Published:** 2021-04-05

**Affiliations:** 1 Centro Universitário FMABC Santo André SP Brasil Professor da Disciplina de Urologia do Centro Universitário FMABC, Santo André, SP, Brasil

Prostate-specific antigen (PSA) is a protease mainly produced by prostatic epithelium; due to the increase of its production by prostatic cancer cells, PSA has been used as a marker in the diagnosis and follow-up of prostate cancer (1).

PSA production is under testosterone influence. Thus, a finding of low PSA could predict a low level of testosterone. Amadi and cols. (2), in a well-designed trial, evaluated the hypothesis that PSA could be a marker of male hypogonadism. The authors prospectively compared a population of male partners from infertile couples, aged 35-44 years, divided in two arms: first group with a mean total testosterone level of 5.68 nmol/L and symptoms of loss of libido and/or erectile dysfunction on ADAM questionnaire (male hypogonadism group); the second group with a mean total testosterone level of 19.23 nmo/L and no sexual complaints on the questionnaire. They found that both total and free PSA showed good sensitivity for predicting male hypogonadism (MH), but poor specificity, whereas free PSA indicated a higher specificity for the diagnosis than total PSA (42% vs. 37%). The authors concluded that low specificity makes free and total PSA a poor marker for MH (2).

However, Rastrelli and cols. (3) came to a different conclusion. They followed almost 3,000 patients with no prior history of prostate disease and PSA lower than 4 ng/dL and reported higher specificity for male hypogonadism diagnosis (55%). This study focused on an older population (mean age of 52.5 years) compared to the group analyzed by Amadi and cols. Rastrelli and cols. concluded that PSA could be a marker for male hypogonadism.

The exact relationship between testosterone and PSA is still unclear. For example, Mustafa and cols. (4) found no correlation between PSA and testosterone levels in healthy men with PSA < 4 ng/dL. Also, Shukla and cols. found no relationship between testosterone and PSA levels in healthy males and even in men with partial androgen deficiency (5).

Those different findings can be explained by the fact that PSA levels are not fully testosterone-dependent in all situations. Older men with enlarged prostate have higher PSA levels and other prostate diseases, such as infection and cancer, might increase circulating PSA rapidly and independently of testosterone levels (1). In a systematic review and meta-analysis, Kim and cols. (6) found that intramuscular testosterone replacement therapy showed a significant change in PSA levels. Same results were not observed with oral or topical therapy. In a subgroup of patients without benign prostate hyperplasia (BPH) they also found a significant correlation between testosterone and PSA.

Morgentaler and Traish (7) proposed that prostate androgen-receptors respond producing PSA until certain level of circulating testosterone. After that, an increase on testosterone level does not correspond to a raise on PSA, as the receptors become insensitive or saturated. Therefore, a permanent correlation between testosterone and PSA probably doesn’t occur.

On the other hand, PSA levels in young males are very low and stable. Pazeto and cols. (8) followed about 93,000 healthy men who underwent PSA tests between the ages of 21-39 years. The mean value ranged from 0.66 ng/mL (at age 22) to 0.76 ng/mL (at age 39) (mean 0.73 ng/mL). Authors pointed to a PSA increase of 0.0055 ng/mL each year. Therefore, the finding of a low PSA value in man does not necessarily represent a state of low testosterone. Amadi and cols. (1) reported that a total PSA value of < 0,74 ug/l could predict MH and this result can be considered normal in many young men.

A biological marker or biomarker is, by definition, a molecular, histologic, radiological, or physiological characteristic that is objectively measured and evaluated as an indicator of normal biological processes, pathogenic processes, or pharmacologic responses to a therapeutic intervention (9). Also, a reliable marker must have high sensitivity and a high specificity. In conclusion, although there is a correlation between testosterone and PSA in many clinical situations and populations, PSA cannot be used as a reliable marker of MH.
